# Artificial Intelligence–Powered Quantification of Flortaucipir PET for Detecting Tau Pathology

**DOI:** 10.2967/jnumed.125.269636

**Published:** 2025-11

**Authors:** Hye Bin Yoo, Seung Kwan Kang, Seong A. Shin, Daewoon Kim, Hongyoon Choi, Yu Kyeong Kim, Dahyun Yi, Min Soo Byun, Dong Young Lee, Jae Sung Lee, Alzheimer’s Disease Neuroimaging Initiative, Korean Brain Aging Study for the Early Diagnosis and Prediction of Alzheimer’s Disease Research Group

**Affiliations:** 1 Institute for Data Innovation in Science, Seoul National University, Seoul, Korea;; 2 Brightonix Imaging Inc., Seoul, Korea;; 3 Interdisciplinary Program of Bioengineering, Seoul National University, Seoul, Korea;; 4 Artificial Intelligence Institute, Seoul National University, Seoul, Korea;; 5 Department of Nuclear Medicine, Seoul National University College of Medicine, Seoul, Korea;; 6 Department of Nuclear Medicine, Seoul Metropolitan Government–Seoul National University Boramae Medical Center, Seoul, Korea;; 7 Institute of Human Behavioral Medicine, Seoul National University Medical Research Center, Seoul, Korea;; 8 Department of Psychiatry, Seoul National University College of Medicine, Seoul, Korea;; 9 Department of Neuropsychiatry, Seoul National University Hospital, Seoul, Korea; and; 10 Convergence Research Center for Dementia, Seoul National University Medical Research Center, Seoul, Korea

**Keywords:** flortaucipir PET, AI-powered image processing, hyperphosphorylated tau, Braak stage, automatic spatial normalization

## Abstract

We developed and evaluated an artificial intelligence (AI)–powered approach for easier quantification of tau PET uptake without requiring structural MR to aid earlier tracking of Alzheimer disease (AD). **Methods:** We implemented a deep neural network model that normalizes ^18^F-AV1451 (tau) PET images to a standard template without requiring MR, using transfer learning from a model pretrained on amyloid PET. This model was integrated into an MR-free pipeline for tau PET quantification and validated on external dataset (Alzheimer Disease Neuroimaging Initiative). We examined correlations between model-derived tau uptake estimates and cognitive measures, including AD stage and episodic memory performance (*n* = 666). Longitudinal analyses were conducted to assess whether baseline tau deposition predicted future cognitive decline (*n* = 168). **Results:** The AI-powered pipeline achieved robust performance with intraclass correlation coefficients exceeding 0.97 for regional uptake estimation compared with MR-based ground truth. We also showed that the tau deposition in metatemporal regions was significantly correlated with Mini-Mental State Examination and Montreal Cognitive Assessment scores. Elevated tau PET uptake in the entorhinal cortex and inferior temporal gyrus predicted future cognitive decline. **Conclusion:** The proposed AI-powered pipeline enhances the clinical accessibility of tau PET by reducing scan costs and streamlining the uptake quantification, achieving high performance without requiring structural MR. We further demonstrated that the pipeline yields cognitively relevant outcome measures for early diagnosis and monitoring of AD progression to aid more personalized treatment strategies targeting AD biomarkers.

## Visual Abstract

**Figure:**
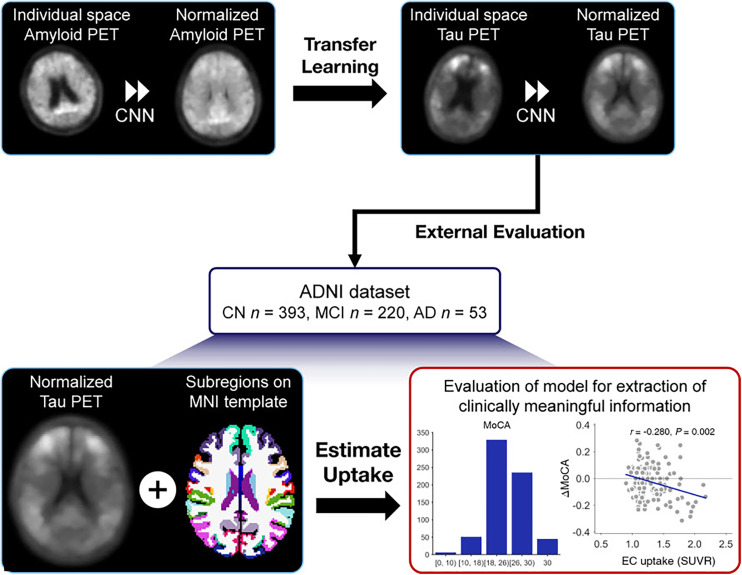

Two characteristic biomarkers of Alzheimer disease (AD) are amyloid-β plaques and neurofibrillary tangles composed of hyperphosphorylated tau protein (amyloid and tau) (1). Amyloid pathologies occur earlier than the symptomatic stage of AD, whereas the propagation of tau pathologies is more closely associated with neuronal loss and subsequent cognitive deterioration (2–5). Thus, brain mapping of tau deposition using PET can aid earlier monitoring of AD pathologies and better observation of treatment responses as in the clinical trial of donanemab, an antiamyloid monoclonal antibody (6). Tau PET is now incorporated into the revised AD diagnosis and staging criteria (National Institute on Aging/Alzheimer’s Association guidelines) (1). Flortaucipir (^18^F-AV1451), a radiopharmaceutical for tau PET approved by the U.S. Food and Drug Administration and European Medicines Agency, is therefore expected to gain greater clinical utility (7).

Although tau PET offers significant diagnostic values via expert visual assessment and quantitative uptake analysis (8), its accessibility remains limited because of difficulties in preprocessing, such as spatial normalization. In addition, an accurate uptake quantification is limited without structural MR images that provide anatomic details for the PET images. We therefore aimed to develop an artificial intelligence (AI)–powered pipeline for tau PET quantification that clinicians can easily use without the need for complex preprocessing steps and paired structural MR images, continuing from our groups’ previous studies (9,10).

This paper presents a novel AI-powered pipeline for MR-free processing of tau PET images. We implement a robust model using a model pretrained on amyloid PET and amplifying the number of training inputs via intensive data augmentation. Following the guidelines for AI-powered nuclear medicine (11), we validate the pipeline using a multisite external dataset from the Alzheimer Disease Neuroimaging Initiative and demonstrate that the proposed pipeline supports more streamlined clinical workflows by providing accurate tau PET quantification without MR and producing uptake estimates that are relevant to cognitive decline.

## MATERIALS AND METHODS

This study was approved by the Institutional Review Boards of Seoul National University Hospital and Seoul National University–Seoul Metropolitan Government Boramae Medical Center in Seoul, Korea, and was conducted according to the current version of the Declaration of Helsinki. We collected written informed consent forms from all participants.

### Datasets

The proposed model was developed using ^18^F-AV1451 PET (tau PET) and the paired structural MR images in the Korean Brain Aging Study for the Early Diagnosis and Prediction of Alzheimer Disease (KBASE) database (12). A cohort of 180 native Koreans (Supplemental Table 1; supplemental materials are available at http://jnm.snmjournals.org) from multiple hospitals was selected for training the deep neural network, which performs spatial normalization of tau PET images. We used the ^18^F-AV1451 PET and structural MR images of Alzheimer Disease Neuroimaging Initiative (ADNI3) database (13) for external validation and testing of the model. This cohort included 1,134 tau PET and MR image pairs acquired from 788 unique subjects. A subset of 146 tau PET and MR images, screened for severe image artifacts and extreme cerebral atrophy, were randomly selected for external validation steps. For external testing, we used PET images, and the corresponding clinical scores were measured at the earliest date for the 666 subjects (Table 1). We also used data from 168 subjects who had multiple sessions of PET scans and cognitive assessments (longitudinal data). Details on KBASE and ADNI datasets are in the supplemental materials.

**TABLE 1.** Demographics of ADNI Subjects (*n* = 666)

Diagnosis	*n*	Age (y)	Ratio of male:female
CN	393	72.50 ± 8.12	157:236
Mild cognitive impairment	220	73.05 ± 7.76	125:95
AD dementia	53	74.74 ± 8.21	34:19

### Training Deep Neural Network

The convolutional neural network model (cascaded U-net (14)) is trained to create nonlinear deformation fields in 3-dimensional space required for spatial normalization using PET images as input (Supplemental Fig. 1). These deformation fields transform individual MR images in native space to fit the MNI template (ICBM152NLin2009 (15)). Network training optimizes the performance of spatial normalization by maximizing the similarity between the standardized MR image and the template. The fully trained model can use tau PET images as the only input for their normalization without paired MR images (MR-free). Details on model training are in the supplemental materials.

To overcome the limited number of tau PET images available for training, we applied a transfer learning technique to use a model pretrained for amyloid PET. We previously developed a deep neural network model for the automatic spatial normalization as originally introduced previously (9). Transfer learning was conducted by finely tuning the network parameters. We increased the number of training inputs (data augmentation) by dynamically generating random changes in the images, such as adding noise and applying transitions or rotations, which prevented overfitting and enhanced the model’s robustness. Technical details including optimization for model training (16) are summarized in the supplemental materials.

### Evaluation of Model in Uptake Quantification

#### Comparison Between Pipelines

**FIGURE 1.**
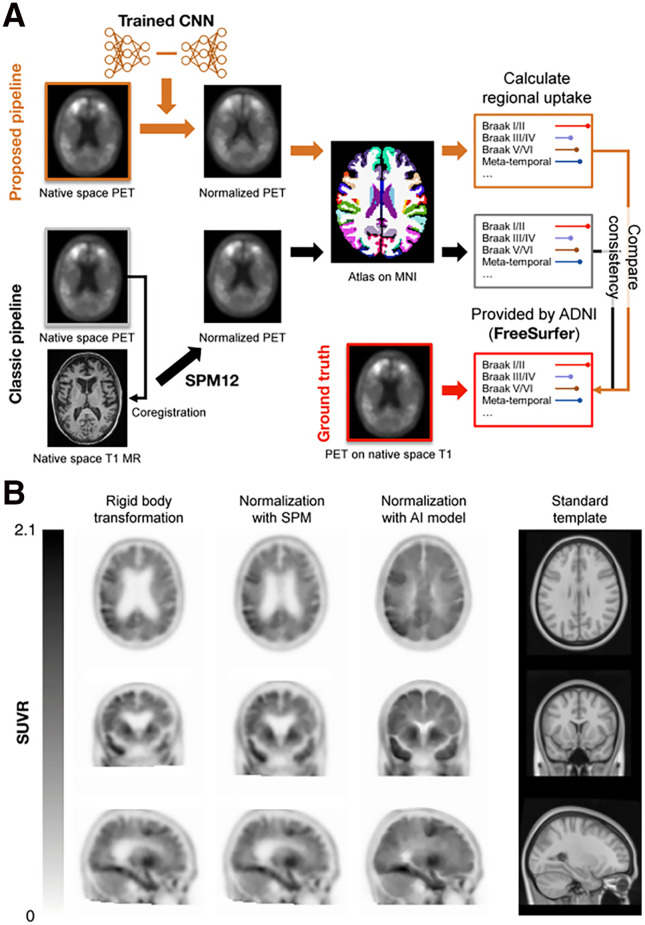
Illustration of workflows in model training and PET quantification pipelines and results of spatial normalization of ^18^F-AV1451 PET image. (A) Procedures of proposed (MR-free) and classic (MR-based) pipelines for quantifying regional tau PET uptake. (B) Input image with rigid body transition applied, normalized using Statistical Parametric Mapping (SPM12), and normalized using our AI model, which are compared with standard template.

For the external validation step (validation dataset from ADNI, *n* = 146), we used the proposed MR-free, AI-powered pipeline, which uses PET images as the only input for spatial normalization and tau PET quantification, and the classic MR-based pipeline, which uses both PET and MR images for the same outcome. Both pipelines were compared with the ground truth values of tau uptake provided by ADNI, which originate from PET images coregistered to T1 MR on native space using FreeSurfer (http://surfer.nmr.mgh.harvard.edu). Figure 1A shows each pipeline’s workflow. The classic pipeline used Statistical Parametric Mapping (http://www.fil.ion.ac.uk/spm) for MR/PET coregistration, normalization, and uptake calculation, whereas the proposed pipeline used Statistical Parametric Mapping only for calculating SUV ratios (SUVRs).

#### Regions of Interest (ROIs)

We quantified regional tau PET uptake in Braak stage ROIs parcellated in the standard MNI template (Supplemental Table 2). Braak stages stratify the tau pathology severity in AD using the patterns of neurofibrillary tangle deposition (17). These stages progress from the entorhinal cortex (EC) and perirhinal cortices (stages I and II) to the medial temporal lobe (III and IV) and finally to wider neocortical regions (V and VI). The metatemporal region (18), known to reflect tau deposition at various stages of cognitive decline in the elderly (19), was analyzed separately (Supplemental Table 2). The SUVRs were calculated by dividing the PET activity concentration in each ROI by that of the cerebellar gray matter.

Tau pathology appears to initiate in the lateral EC (earliest Braak stage regions including the transentorhinal cortex) and subsequently spread, with the inferior temporal gyrus (ITG) likely functioning as a hub driving extensive tau propagation (2). On the basis of these findings, we further analyzed regional uptake in EC and ITG to assess the association between tau PET uptake and cognitive measures. Regions were defined using the Desikan–Killiany atlas (20,21).

### Evaluation of Model to Provide Clinically Relevant Information

#### Cognitive Measures

We used the Clinical Dementia Rating (CDR) global scale (0–3 points, with 3 being the worst) for gauging clinical severity related to AD (22): cognitively normal (CN; CDR, 0), mildly cognitively impaired (CDR, 0.5), and AD (CDR, ≥0.5). For some analyses, only scans from subjects with a CDR of 0 were included. General cognitive functions were assessed using the Mini-Mental State Exam (MMSE, 0–30 points, with 30 being the best), with an MMSE of 25 being the threshold for CN (23). For more sensitive detection of early impairments, Montreal Cognitive Assessment (MoCA, 0–30 points, with 30 being the best) was used, with a MoCA of 26 being the normal threshold (24).

Episodic memory performance was analyzed using quantified cognitive processes developed by Embic Corp. (25,26). This measure is derived from wordlist memory tests and uses the hierarchical Bayesian cognitive processing model to account for variations in wordlist presentation and encoding (27). Memory performance is expressed as a latent probability (0–1) of recall across tasks. We evaluated 4 measures of episodic memory: mean immediate and delayed recall probabilities for task 1 and recall from nondurably and durably stored memory for any task (details provided in the supplemental materials (28,29)).

### Statistical Analyses

We correlated SUVRs obtained using 2 pipelines, with the ground truth originating from native space, and calculated intraclass correlation coefficients (supplemental materials) to evaluate the robustness of the proposed pipeline. To verify if the AI-powered pipeline produces cognitively relevant results, we correlated SUVR with cognitive measures corresponding to the PET images acquired on the earliest date available. We selected a subset of CN subjects (CDR, 0) to examine whether tau deposition in clinically normal individuals is linked to lower functions. Lastly, we analyzed longitudinal PET scans from 168 subjects to explore whether baseline tau deposition predicts later cognitive changes.

## RESULTS

### PET Uptake Quantification Accuracy

**FIGURE 2.**
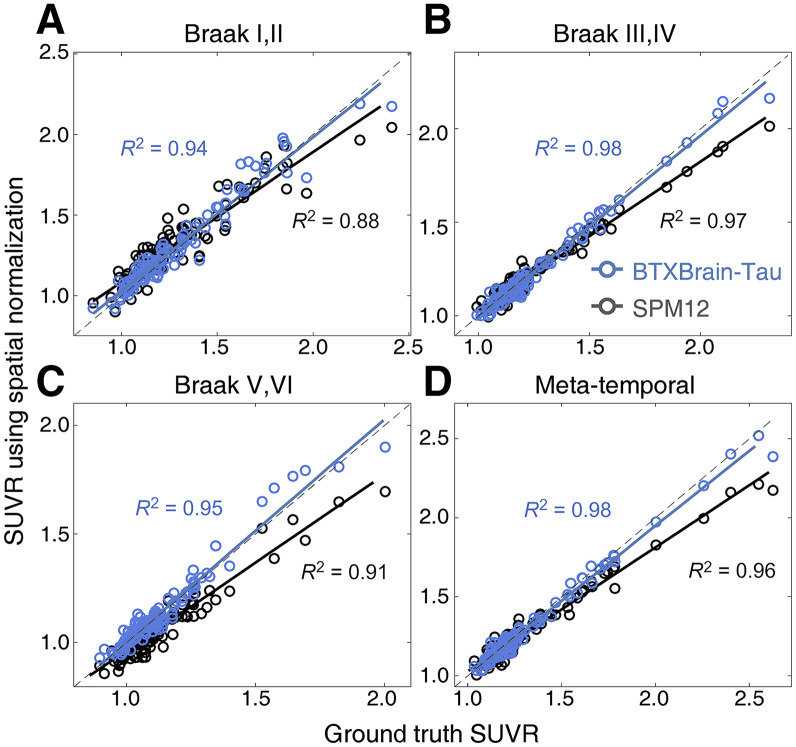
Evaluation of model performance. (A) Braak I and II. (B) Braak III and IV. (C) Braak V and VI. (D) Metatemporal. *x*-axis represents ground truth SUVR estimated in native space using FreeSurfer, whereas *y*-axis is estimated in MNI template using either classic (MR-based, black lines) or AI-powered (MR-free, blue lines) pipeline. BTXBrain-Tau = AI-powered software from Brightonix Imaging. SPM12 = Statistical Parametric Mapping version 12.

We visually inspected the normalized PET images and found that those created by our model retained more morphologic details that fit the template, compared with the MR-based normalization using Statistical Parametric Mapping (Fig. 1B). We verified that the tau PET quantification using the AI-powered pipeline for our model outperforms the classic pipeline in terms of consistency with the MR-based PET quantification using FreeSurfer in native space (Fig. 2; Table 2; Supplemental Fig. 2). The supplemental materials contain details on the results.

**TABLE 2.** Intraclass Correlation of SUVR Quantifications Compared with Ground Truth

Region	SPM12	Proposed AI-based
Braak I and II	0.923 (0.895–0.949)	0.972 (0.959–0.980)
Braak III and IV	0.963 (0.947–0.975)	0.990 (0.985–0.993)
Braak V and VI	0.942 (0.917–0.960)	0.975 (0.964–0.983)
Metatemporal	0.958 (0.940–0.971)	0.991 (0.987–0.994)

SPM12 = Statistical Parametric Mapping version 12.

95% CIs are in parentheses.

### Clinical Association Between PET Uptake and Cognitive Measures

#### AD Stages

**FIGURE 3.**
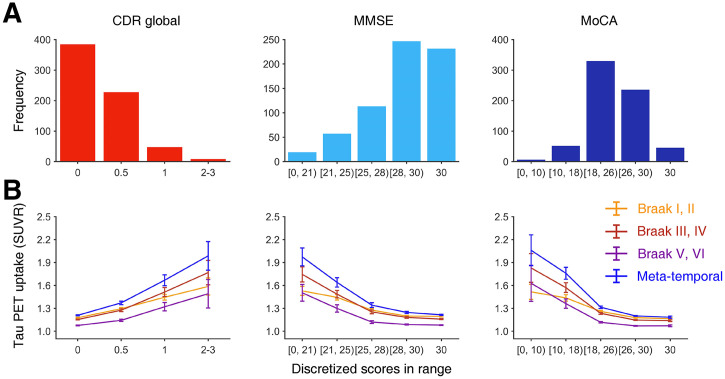
Distribution of discretized clinical scores of functional impairments and corresponding PET uptake in SUVR (*n* = 666). (A) Histogram of score ranges in CDR global, MMSE, and MoCA. (B) PET uptake for Braak stage and metatemporal ROIs. Line plots indicate mean ± SE.

Figure 3A shows the histogram of the discretized clinical scores in all subjects who have ^18^F-AV1451 PET data and 3 clinical scores (*n* = 666 corresponding to clinical scores measured on the earliest date given multiple scans). Figure 3B shows that regional tau PET uptake varies by clinical stages. Bivariate correlation analyses showed that MMSE and MoCA scores have significant negative correlation with regional tau PET uptake in any Braak stage and metatemporal region (*n* = 666, *r* ≤ −0.421, corrected *P* < 0.001) so that for higher performers tau uptake is lower (similar patterns in the temporal and parietal subregions; Supplemental Fig. 3).

We focused on CN subjects (CDR, 0) to see if the tau PET uptake still correlates with cognitive performance (MMSE and MoCA) within the nonclinical population. In total, 384 scans were included in the analyses (CDR global; Fig. 3A). One-sided bivariate correlation analysis revealed that, among all ROIs, higher tau PET uptake was not correlated with a lower MMSE score (*r* ≥ −0.078, uncorrected *P* ≥ 0.065) but was correlated with a lower MoCA score specifically in the metatemporal region in CN subjects (*r* = −0.147, *P* = 0.015, Bonferroni-corrected for 8 cases). Negative correlation in Braak III and IV regions did not survive multiple correction (corrected *P* = 0.057). No other regions showed significant results (*r* ≥ −0.080, uncorrected *P* ≥ 0.059). Table 3 reports the results of correlation analysis on clinical scores.

**TABLE 3.** Correlations Between Tau Uptake and Clinical Scores*

	All subjects (*n* = 666)		CDR of 0 (*n* = 384)	
Region	MMSE	MoCA	MMSE	MoCA
Braak I and II	−0.433 (*P* < 0.001)	−0.421 (*P* < 0.001)	−0.030 (*P* = 0.278)	−0.075 (*P* = 0.072)
Braak III and IV	−0.538 (*P* < 0.001)	−0.527 (*P* < 0.001)	−0.056 (*P* = 0.136)	−0.125 (*P* = 0.007)
Braak V and VI	−0.455 (*P* < 0.001)	−0.437 (*P* < 0.001)	0.004 (*P* = 0.533)	−0.080 (*P* = 0.059)
Metatemporal	−0.549 (*P* < 0.001)	−0.544 (*P* < 0.001)	−0.077 (*P* = 0.065)	−0.147 (*P* = 0.002)

* 2-sided Pearson correlation, *P* < 0.006 is significant.

#### Episodic Memory Performance

**FIGURE 4.**
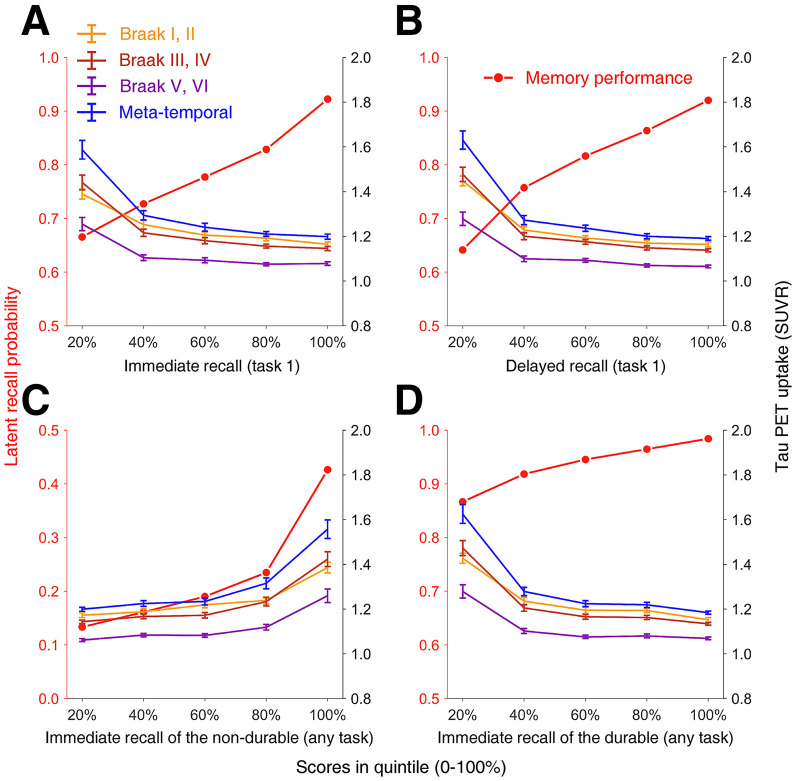
Episodic memory performance correlated with tau PET. (A) Immediate recall (task 1). (B) Delayed recall (task 1). (C) Immediate recall of nondurable (any task). (D) Immediate recall of durable (any task). Memory performance quantified as probability (0–1, left *y*-axis in red) was discretized into quintiles (20%–100% of *n* = 666). Tau uptake is in SUVR (right *y*-axis in black). Note that nondurable recall values range from 0 to 0.5.

We stratified latent episodic memory performance by quintile scores (20%–100% of *n* = 666) and matched each group’s mean regional SUVR (Fig. 4). For all but the probability of immediate recall of nondurably stored episodic memory, the higher tau PET uptake was related to the lower episodic memory performance (higher in 20%). This pattern is consistent with the descriptive report provided by ADNI (26). Similar patterns are found in the temporal and parietal subregions (Supplemental Fig. 4).

We analyzed CN subjects (CDR, 0) to correlate tau deposition with earlier episodic memory dysfunctions. Two-sided bivariate correlation analyses found that after Bonferroni adjustment for 16 comparisons, tau PET uptake of Braak III and IV regions was significantly correlated with all 4 measures of episodic memory (corrected *P* ≤ 0.049). Correlation patterns were similar in the metatemporal region, except for the immediate recall performance of nondurably stored episodic memory (corrected *P* = 0.071). Tau deposition in Braak I and II regions that starts earlier than in Braak III and IV regions was not significantly correlated with episodic memory performance except for the delayed recall probability for task 1 (corrected *P* = 0.006, higher deposition relates to lower performance). Table 4 demonstrates the results of correlation analysis on episodic memory performance.

**TABLE 4.** Correlations Between Tau Uptake and Episodic Memory Performance of CN Subjects with CDR of 0*

Region	Immediate recall 1	Delayed recall 1	Immediate recall of nondurable	Immediate recall of durable
Braak I and II	−0.088 (*P* = 0.084)	−0.181 (*P* < 0.001)	0.069 (*P* = 0.179)	−0.116 (*P* = 0.023)
Braak III and IV	−0.194 (*P* < 0.001)	−0.274 (*P* < 0.001)	0.151 (*P* = 0.0031)	−0.234 (*P* < 0.001)
Braak V and VI	−0.090 (*P* = 0.080)	−0.172 (*P* < 0.001)	0.128 (*P* = 0.012)	−0.143 (*P* = 0.005)
Metatemporal	−0.207 (*P* < 0.001)	−0.287 (*P* < 0.001)	0.145 (*P* = 0.004)	−0.244 (*P* < 0.001)

* 2-sided Pearson correlation, *P* < 0.0031 is significant.

#### Prediction of Cognitive Changes Using Baseline Tau Uptake

**FIGURE 5.**
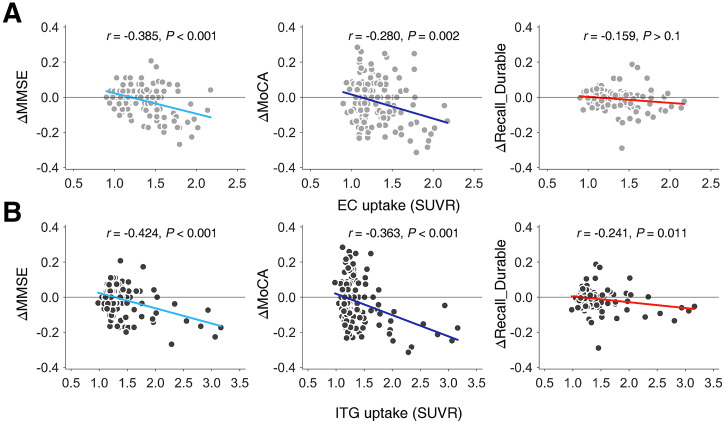
Baseline tau uptake predicts later changes in AD clinical measures. Changes (Δ) in MMSE, MoCA, and probability of recall for durably stored episodic memory are shown for EC (A) and ITG (B). Line plots indicate linear fits.

We performed a longitudinal analysis on a subset with multiple scans (*n* = 168). We aimed to investigate if the tau PET uptake measured in earlier scans predicts a decline in MMSE, MoCA, and episodic memory performance in recall probability of the durable memory, focusing on the EC where tau deposition initiates, and the ITG where propagation of tau deposition is triggered. We correlated the regional tau PET uptake acquired at the earliest date (baseline) available with the ratios of changes in clinical measures ([Post − Pre]/[Pre + 0.1]), where Post indicates the scores measured at the latest available date and Pre the earliest. The exam dates of cognitive measures were closely matched to the scan dates by minimizing the absolute difference. The follow-up durations ranged from 221 to 1,007 d, with the most common intervals centered around 1 y (*n* = 56, 34%) and 2 y (*n* = 38, 23%). Outliers with magnitudes of changes exceeding 0.5 were excluded (*n* = 165). Correlation analyses found that the higher baseline tau deposition in EC and ITG predicts the decrease in MMSE and MoCA later in life (Fig. 5, *r* ≤ −0.280, Bonferroni-corrected *P* ≤ 0.002 for 6 cases). However, the uptake in EC was not significantly related to the later decline in episodic memory performance (*r* = −0.159, corrected *P* = 0.251), whereas that in ITG was (*r* = −0.241, corrected *P* = 0.011).

## DISCUSSION

We developed a neural network model that processes ^18^F-AV1451 PET images for automatic spatial normalization to aid uptake estimation. The proposed method streamlines tau PET uptake estimation in clinical practice. Trained on an Asian population dataset and validated on the external ADNI3 dataset, the model produced tau uptake estimates consistent with ground truth. AI-powered estimates of tau deposition in Braak stage regions showed significant correlations with clinical measures used for AD staging and episodic memory performance (30). Tau uptake in the Braak III and IV regions and metatemporal region (18) was associated with lower MoCA scores and episodic memory in CN subjects. Uptake in ITG predicted future cognitive decline in ADNI3, consistent with previous studies (2,30–32).

Tau PET uptake has shown more prominent clinical relevance than amyloid PET uptake (33). Studies clarifying the separate pathologic courses of tau and amyloid deposition in AD indicate that the progression of tau pathologies demonstrates a typical regional propagation pattern, in contrast to the diffuse deposition of amyloid (5,31,32); that global amyloid deposition precedes widespread tau dispersion (2,3); that the interaction between 2 pathologies leads to rapid and devastating disease progression (2,30); and that tau deposition near the medial temporal cortex relates to earlier cognitive dysfunctions such as episodic memory loss (3,31) as demonstrated in this study. We further observed that, although tau deposition in ITG was significantly correlated with cognitive measures, its baseline levels remained clustered at lower values across a broader range (particularly for MoCA), whereas that in EC was more widely distributed across the same range. This pattern aligns with the Braak staging and current models of the spatiotemporal progression of preclinical AD. In sum, tau deposition in EC may serve as a sensitive biologic marker of earlier neurodegenerative changes, whereas increased tau in metatemporal ROIs including ITG may represent a clinical inflection point signaling the transition to accelerated pathologic progression (2,5,30–32). Incorporating tau PET may provide clinicians with an effective opportunity to timely intervene in the progression of cognitive impairments.

We demonstrate that the proposed pipeline powered by an AI model for spatial normalization provides clinically meaningful information through accurate tau PET quantification using simplified processing steps without MR dependency. Rather than serving as a standalone diagnostic agent, our tool assists human experts’ interpretations and more informed diagnostic decisions (8). We expect that using the proposed pipeline will expand the routine use of tau PET, which will support tracking biomarkers associated with cognitive decline that precedes symptomatic progression of AD (2), predicting the trajectory of AD-related cognitive decline (1), and identifying the window of therapeutic intervention for improving prognosis (6). Recent advances in antiamyloid monoclonal antibody treatment (i.e., lecanemab and donanemab) have shown some success in slowing disease progression (34,35) and in reducing both amyloid levels detected by PET and tau levels in cerebrospinal fluid. Accordingly, tau PET could be effective for monitoring the treatment effects. Our AI-powered pipeline links regional tau levels in the metatemporal region with clinically relevant cognitive dysfunctions, potentially increasing the specificity in detecting medication efficacy.

One potential advance in clinical practice is to use our tool for normative modeling of tau PET uptake. Normative modeling allows for the assessment of a subject’s functional state compared with a reference or a general control. Higher clinical accessibility achieved with our AI-powered tool will aid collection of tau PET data from more diverse populations, which is important for building a robust normative model. Further studies should aim to refine AI-powered diagnostic tools to integrate biomarker quantification from multiple radiotracers such as ^18^F-AV1451, ^18^F-florbetaben, and ^18^F-FDG to help clinicians minimize biases from different quantification models. Using large datasets with multimodal images, such as those from ADNI, we can also test the model’s ability to predict future functional decline of a subject based on the current state. We should also acknowledge limitations in the tool’s generalizability to non-AD tauopathies and cases with pronounced morphologic changes, such as severe atrophy, which could be addressed by incorporating a more diverse set of training inputs. In addition, the current pipeline does not support inverse transformation from the MNI space to native PET space that enables extracting SUVRs without interpolation. Future implementations will address this by accounting for individual variability in uptake after AI-powered normalization.

## CONCLUSION

We proposed an AI-powered pipeline that enhances the clinical accessibility of ^18^F-AV1451 PET via simplified workflows for tau quantification. We validated its performance and robustness using external datasets, showing that the estimated tau deposition is clinically meaningful through correlations with cognitive measures. The proposed pipeline can aid monitoring cognitively normal individuals at risk for AD and evaluating the effects of antiamyloid medicines.

## DISCLOSURE

We were supported by Korea Dementia Research Project (No. HU23C014000) via Korea Dementia Research Center, funded by the Ministry of Health & Welfare and Science and ICT; the Global-LAMP Program (No. RS-2023-00301976); and the K-Brain Project (No. RS-2023-00264160) of the National Research Foundation of Korea (NRF). This study also used the KBASE database, supported by the New Faculty Startup Fund from Seoul National University; NRF grants (Nos. RS-2022-00165636 and 2014M3C7A1046042); the Korea Health Technology R&D Project through the Korea Health Industry Development Institute (Nos. HI18C0630 and HI19C0149); and a grant from the National Institute on Aging, USA (U01AG072177). No other potential conflict of interest relevant to this article was reported.

## Notes

KEY POINTS **QUESTION:** Can our AI-powered pipeline for tau PET quantification achieve clinically relevant results without structural MR? **PERTINENT FINDINGS:** We propose an AI-powered MR-free pipeline that demonstrates robust agreement with MR-based quantification and correlates with cognitive measures. **IMPLICATIONS FOR PATIENT CARE:** The proposed pipeline enhances the clinical accessibility of tau PET and facilitates monitoring of AD progression.
